# Vertical nanoscale strain-induced electronic localization in epitaxial La_2/3_Sr_1/3_MnO_3_ films with ZrO_2_ nanopillar inclusions

**DOI:** 10.1186/s40580-023-00382-6

**Published:** 2023-07-28

**Authors:** Yuze Gao, Manuel A. Roldan, Liang Qiao, David Mandrus, Xuechu Shen, Matthew F. Chisholm, David J. Singh, Guixin Cao

**Affiliations:** 1 Materials Genome Institute, Shanghai University, Shanghai, 200444 China; 2 Materials Science and Technology Division, Oak Ridge National Laboratory, Oak Ridge, TN 37831-6056 USA; 3 Departamento Fisica Aplicada III, Facultad de Ciencias Fisicas, Universidad Complutense de Madrid, 28040 Madrid, Spain; 4 Center for Nanophase Materials Sciences, Oak Ridge National Laboratory, Oak Ridge, TN 37831 USA; 5 National Laboratory for Infrared Physics, Shanghai Institute of Technical Physics, Chinese Academy of Sciences, Shanghai, 200083 China; 6 Department of Physics and Astronomy, University of Missouri, Columbia, MO 65211-7010 USA; 7 Zhejiang Laboratory, Hangzhou, 311100 China

## Abstract

Unusual electrical transport properties associated with weak or strong localization are sometimes found in disordered electronic materials. Here, we report experimental observation of a crossover of electronic behavior from weak localization to enhanced weak localization due to the spatial influence of disorder induced by ZrO_2_ nanopillars in (La_2/3_Sr_1/3_MnO_3_)_1−*x*_:(ZrO_2_)_*x*_ (*x* = 0, 0.2, and 0.3) nanocomposite films. The spatial strain regions, identified by scanning transmission electron microscopy and high-resolution x-ray diffraction, induce a coexistence of two-dimentional (2D) and three-dimentional (3D) localization and switches to typical 2D localization with increasing density of ZrO_2_ pillars due to length scale confinement, which interestingly accords with enhancing vertically interfacial strain. Based on the excellent agreement of our experimental results with one-parameter scaling theory of localization, the enhanced weak localization exists in metal range close to the fixed point. These films provide a tunable experimental model for studying localization in particular the transition regime by appropriate choice of the second epitaxial phase.

**Graphical Abstract:**

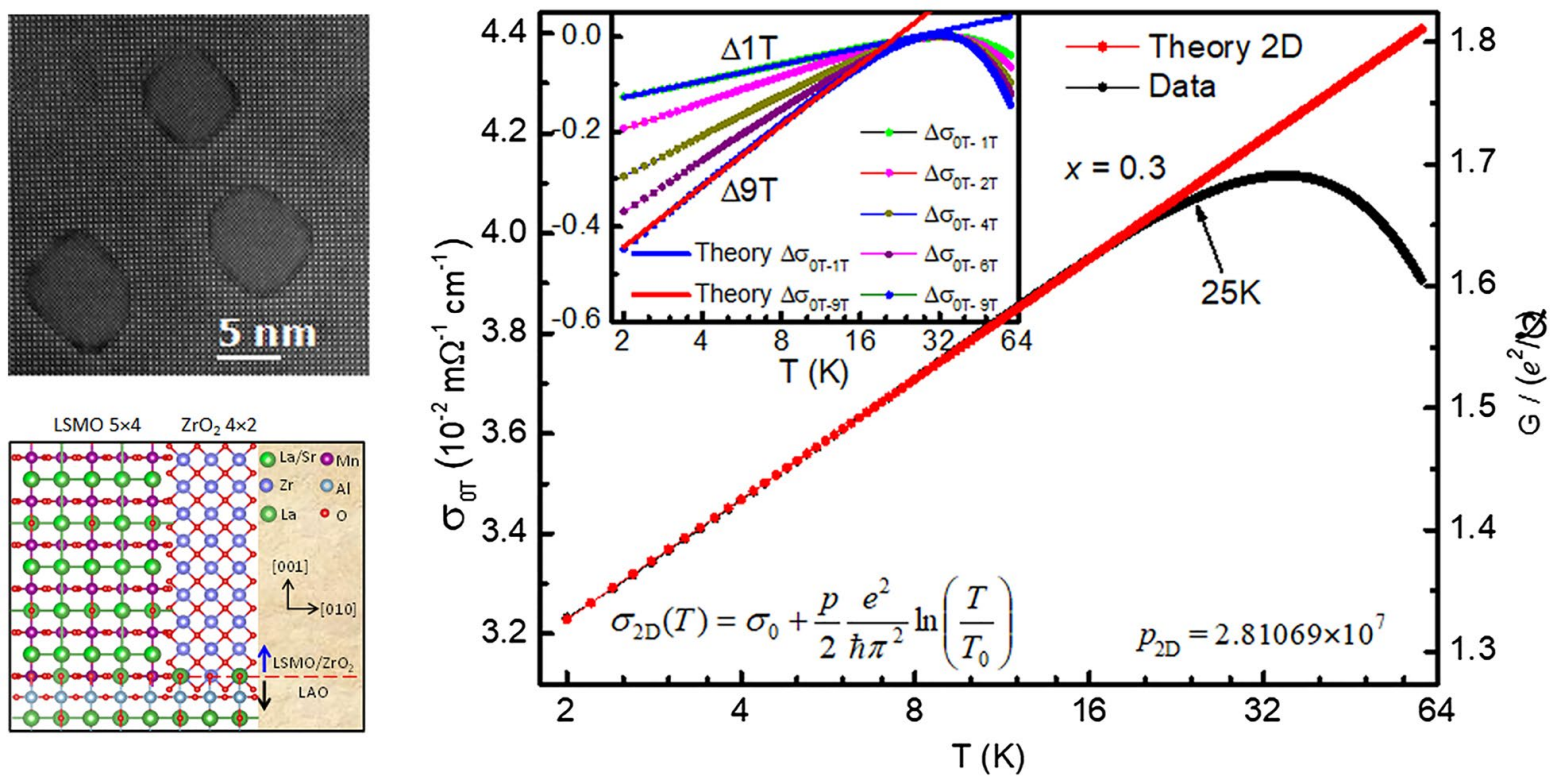

## Introduction

The unusual transport properties that occur in disordered materials are among the most interesting phenomena in condensed matter physics. Disorder generally exists to varying degrees in crystalline matter, ranging from a few impurities or interstitials in an otherwise perfect crystalline host to the strongly disordered limit of alloys or glassy structures [1], depending on the amount and type of disorder as well as dimensionality [2, 3]. Anderson localization (AL) and weak localization (WL) are two important phemomena that occur in disordered electronic systems. Classical Anderson localization, known as strong localization, is multiple scattering interference of the waves due to randomness in the potential, thus altering the nature of the wave functions, [1, 4] usually occurring in one dimentional (1D) and 2D semiconductors or 1D conductors [5]. While, there are many examples of disorder-induced metal–insulator transitions (MIT), unambiguous identification of these as due to classical Anderson localization in 3D remains a controversial subject of discussion in condensed matter physics.[6, 7] Weak localization, to be distinguished from AL, is the precursor effect of AL, that occurs in disordered electronic systems at low temperatures due to back scattering. The effect manifests itself as a positive correction to the resistivity of a metal or semiconductor [8]. Extensive electrical transport measurements show low-temperature weak localization in samples of both high-*T*_*c*_ cuprates and manganites and are understood as quantum interference effects [9–11].

La_2/3_Sr_1/3_MnO_3_ (LSMO) when ferromagnetic is a transport half-metal, meaning that its transport shows metallic conduction in the majority spin channel, and localized behavior due to strong localization, in the minority channel [12–15]. This can be qualitatively understood in terms of the low carrier density, low Fermi energy minority spin electronic structure and the contrasting large Fermi surface, metallic majority spin. Importantly, the above-mentioned properties are extremely sensitive to strain [16, 17]. The emergence of novel electronic states and magnetic reconstructions at the interface of LSMO and another perovskite oxide has been intensively studied, including electronic, magnetic and orbital reconstructions, and interfical magnetism et al [18–22]. To achieve these fascinating properties, the vertical interfical strain (VIS) plays an important role through tunning the interfical orbital, lattice order parameters and resulted film functionality [23] due to its enhanced interfical coupling and tailored vertical 3D strain [24–26]. However, to date, most of the studies on VIS were performed on the materials with a pillar-to-matrix ratio at 1:1 with nanopillars larger than 20 nm [27–29]. To enhance the VIS, rational control over the pillar size and study on the corresponding modification of the functional properties would be of great importance [30].

The strain dependence of the electronic structure, in particular in the minority spin channel for ferromagnetic character, the study of localization and novel electronic states at the interface of LSMO is attractive not only because the effect of 3D vertical disorder on the localization is rarely studied but also because new physics it may bring due to the natural length scale confinement by the nanopillars in the thick films [31]. As far as is known, the study on the effect of small nanopillars on physical properties of matrix, especially the study of spatial nanopillar effect on the electrical localization, has not been explored yet. Here, we report a new approach for localization in successfully grown (LSMO)_1−*x*_:(ZrO_2_)_*x*_ (*x* = 0, 0.2, and 0.3) nanocomposite film system which is based on the 3D spatial disorder resulting from the vertical strain between two different epitaxial structures of LSMO matrix and embedded small ZrO_2_ nanopillars. This specific pillar structure in the matrix provides a natural length scale confinement vertically, determined by the distance between the pillars, which restrict the system approaching low dimensional character with increasing density or size of nanopillars in the film system. Our results demonstrate that the nanopillar structure in the LSMO matrix displays a shallow structural disorder region along the circular interface between ZrO_2_ nanopillar and LSMO due to semi-coherent dislocalization with locally vertical strain, which can be controlled by the size of ZrO_2_ nanopillars. Interestingly, the vertical strain and the interface strongly affect the electronic transport properties of the LSMO matrix induced by the small nano-scale size pillars with accordingly large strained interfical area. We find a critical exponent behavior between 2 and 3D with increasing size of ZrO_2_ in the 3D thin film system and a crossover to a new transition state, which lies between weak localization and Anderson localization. We refer to this enhanced weak localization state as “transition localization”. This induced transition localization is directly related to an enhanced vertical strain at the interface with increasing density and size of ZrO_2_ pillars. Our study paves a way for the functional application on the vertical magnetic devices.

## Experimental details

**A. Thin films synthesis and STEM sample preparations** Epitaxial nanocomposite thin films of (LSMO)_1−*x*_:(ZrO_2_)_*x*_ with *x* = 0, 0.2, and 0.3 of non-magnetic ZrO_2_ nanostructures were grown on LaAlO_3_(LAO) (001) substrates using conventional pulsed laser deposition (PLD). Details of the deposition process are given in Ref [32]. The thickness of the thin films was determined as 45 nm using both TEM and X-ray reflectivity (XRR) measurement [33]. The films were characterized by XRD and by transmission and high-resolution transmission electron microscopy (TEM and HRTEM). Samples for STEM analysis were prepared in cross-sections oriented along the (001) cubic directions and top-view of the (001) surface.

**B. HRTEM and STEM imaging and GPA analysis** The plan-view HAADF study was performed on a Nion UltraSTEM200, equipped with a cold field-emission electron source and a corrector of third- and fifth-order aberrations, operating with an accelerating voltage of 200. Displacement and rotation fields were calculated from high resolution HAADF images using GPA software. GPA is an image-processing routine that is sensitive to small displacements of the lattice fringes in HRTEM/STEM images relative to a reference lattice [34, 35]. The strain tensor ${\varepsilon }_{ij}$ can be obtained by numerical differentiation using the standard relations [34, 36]: $\varepsilon_{ij \, } = \frac{1}{2}\left( {\left. {\frac{{\partial u_{i} }}{{\partial x_{j} }} + \frac{{\partial u_{j} }}{{\partial x_{i} }}} \right)} \right.$. In a similar way the local in-plane rigid body rotation, *ω*_*xy*_, can be determined: $\omega_{xy \, } = \frac{1}{2}\left( {\left. {\frac{{\partial u_{y} }}{{\partial x_{x} }} - \frac{{\partial u_{x} }}{{\partial x_{y} }}} \right)} \right.$, where, the *u(r)* is the local displacement field as a function of position with respect to the reference lattice. For small rotations, the angle is in radians and anti-clock wise positive.

**C. Transport and magnetic property measurements** Evaporated Cr/Au contacts were used for 4-probe resistivity measurements using a Quantum Design PPMS. The resistivity was performed with the magnetic field perpendicular to the film. The magnetization (*M*) was measured in a Vibrating Sample SQUID magnetometer (Quantum Design). All the experimental results were reproducible. No ageing effects were observed.

**D. The correction method for magnetoresistance** We ruled out magnetic effects, i.e. magnetoresistance effects and the electron–electron correlation effects, leaving disorder induced weak localization as an explanation. We corrected the data of MR by shifting the finite field resistance curves by constant values such that the minimum resistivities coincided. This was done because localization is sensitive to the magnetic field, as is the colossal MR effect in La_2/3_Sr_1/3_MnO_3_. In this case, it is necessary to separate out the MR effect in the fitting process. We take the resistivity at the MIT at low temperatures *ρ*_H-MIT_ under various magnetic fields as the basic reference value of *ρ* for the experimental data to calculate the corresponding values of the resistivity according to ${\rho }_{H}\left(T\right)={\rho }_{H-MIT}+\Delta {\rho }_{H}\left(T\right)={\rho }_{0}+{\Delta MR}_{H}+\Delta {\rho }_{H}\left(T\right)$ (0 T ≤ *H* ≤ 9 T)*.* ${\rho }_{0}$ is the value of ${\rho }_{H-MIT}$ at *H* = 0 T, and ${\Delta MR}_{H}$ is the contribution of magnetoresistivity under various magnetic fields at the *T*_MIT_ point at low temperatures. $\Delta {\rho }_{H}\left(T\right)$ is the upturn of the resistivity below *T*_MIT_. At the *T*_MIT_ transiton point, the contributions from weak localization and EEI to the upturn are very weak, so the contribution of Δ*MR*_*H*_ is the only intrinsic quantity of LSMO at the temperature of *T*_MIT_. Then ${\rho }_{H}\left(T\right)$ will be ${\rho }_{H}\left(T\right)={\rho }_{0}+\Delta {\rho }_{H}\left(T\right)$ without ${\Delta MR}_{H}$. In this way, the data which we get from the experimental ones with the magnetoresistivity was ruled out, and the conductivities were obtained from these data.

## Results and discussion

**Fig. 1:**
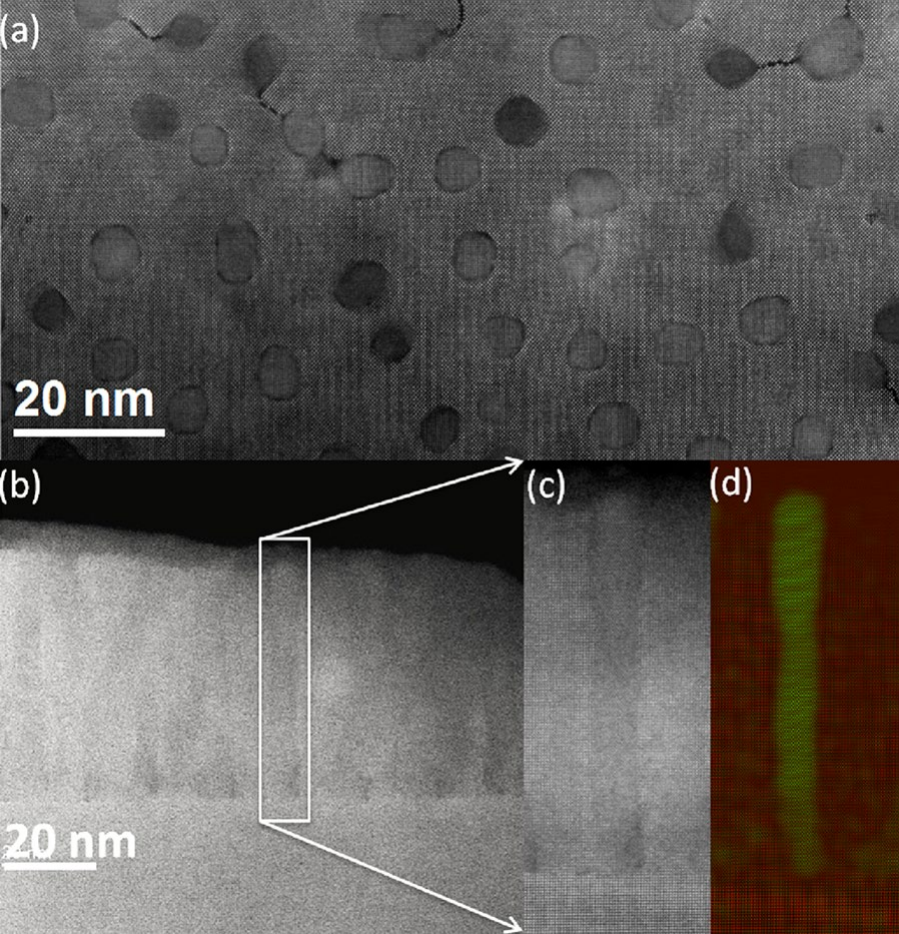
**a** Plane-view high resolution high angle annular dark field (HAADF) image of LSMO thin film with *x* = 0.3 ZrO_2_ precipitatedon LaAlO_3_ (001) substrate. **b** Low mag STEM image of cross-sectioning view of *x* = 0.3 thin film; **c** a relatively high magnification image of a representative single pillar in LSMO; **d** the corresponding false-colored image for the representative single pillar based on the FFT diffractogram

**Fig. 2:**
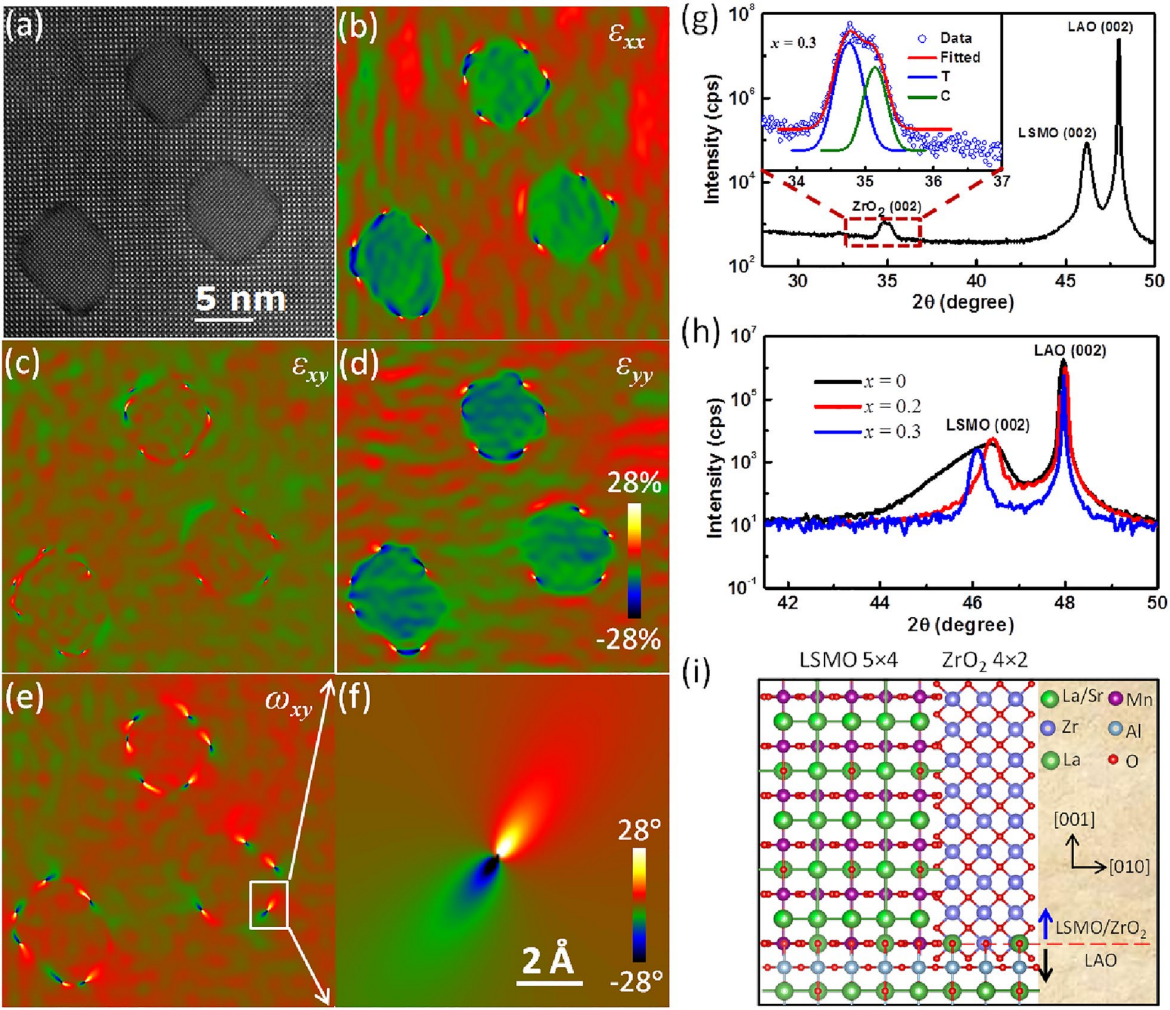
**a** Selected STEM image showing the atomic resoluation lattice of the circular pillars, matrix and the interface; **b** strain component parallel to the surface *ε*_*xx*_, **c** shear-strain map *ε*_*xy*_, **d** perpendicular to the surface *ε*_*yy*_, and **e** the rotation map *ω*_*xy*_ gives the internal rigid-body rotation of the crystallographic lattice obtained by GPA from the HRTEM image of **a**. The scale bar is 5 nm and the colour scale indicates −  28° to + 28° strain and rotation. **f** The magnified view of part of image. The scale bar is 2 Å. **g** XRD *θ*–2*θ* scans of *x* = 0.3 nanocomposite thin film on LAO (001) substrate. Inset shows the overlap (002) peak and the area ratio fitting for cubic and tetragonal phase of ZrO_2_ nanopillars. Here, T represents tetragonal and C represents cubic. **h** The shift of the LSMO (002) peak downward on 2*θ*-scale for (LSMO)_1−*x*_:(ZrO_2_)_*x*_ films with *x* = 0, 0.2 and 0.3, indicating the increase of the *c*-lattice parameter with increasing *x*. **i** Crystallographic model of interface matching

Plane-view high resolution high angle annular dark field (HAADF) image of an LSMO thin film with *x* = 0.3 precipitated ZrO_2_ is shown in Fig. 1a, which displays that ZrO_2_ grows in a circular shape with uniformly spatial distribution in the LSMO matrix. Low magnification scanning transmission electron microscopy (STEM) image of cross-sectioning view (Fig. 1b) reveals that the circular ZrO_2_ pillars penetrates all the way through the LSMO film and distributes uniformly in the LSMO matrix. A representative single pillar was chosen and the perfect pillar structure was observed by the high magnification image (Fig. 1c) and the corresponding false-colored image based on the Fast Fourier Transform (FFT) diffractogram (Fig. 1d). The mean pillar diameters are 4.0 ± 0.6 nm for *x* = 0.2 and 5.1 ± 0.6 nm for *x* = 0.3. The interface between a pillar and the matrix is semi-coherent with misfit dislocations distributed around and along the cylindrical interface. This implies the local strain at the interface, which likely impacts local electronic properties. Figure 2a shows an atomic resolution plane-view STEM image of the (LSMO)_1−*x*_:(ZrO_2_)_*x*_ structure, which shows the clear sharp interface and tiny transformation of structure between the inner and interfical region of the ZrO_2_ pillar between. In order to establish high-resolution electron microscopy as a metrological tool for strain analysis at the atomic level, the displacement field mapping and strain analysis are performed using geometric phase analysis (GPA) [34, 37]. The maps of strain components *ε*_*xx*_, *ε*_*xy*_ and ε_*yy*_ for the area shown in Fig. 2a are displayed in Fig. 2b–d, respectively. The tensile strain in matrix, compressive strain in ZrO_2_ pillars and similar strain in *ε*_*xx*_ and *ε*_*yy*_ are clearly seen. *ε*_*xx*_ and *ε*_*yy*_ show the biggest change, specifical the interfical region, identified as a highly strained area (*ε*_*yy*_ > 20%). While, the observed shear-strain *ε*_*xy*_ is weaker (Fig. 2c). The rotation field mapping of the sample shows rotation between the two structures. The observed phenomena are consistent with the x-ray diffraction (XRD) analysis as explained below, which origins from the mismatch and strain in the epitaxial interface between ZrO_2_ pillars and LSMO matrix.

**Fig. 3:**
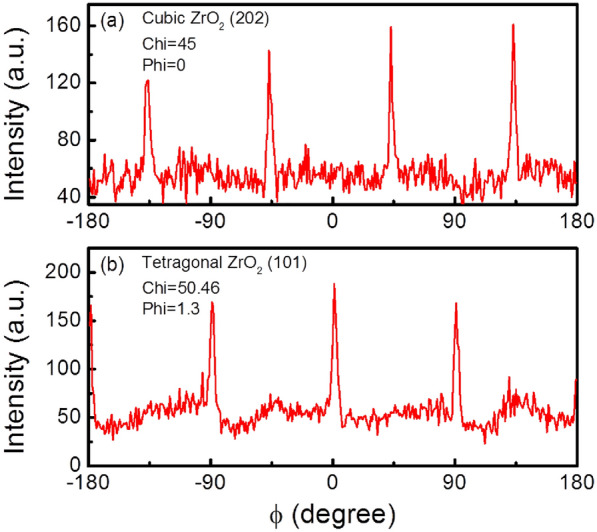
**a** The texture measurements of a (202) reflex for cubic ZrO_2_ phase at Chi = 45 and Phi = 0. **b** The texture measurements of a (101) reflex for tetragonal ZrO_2_ phase at Chi = 50.46 and Phi = 1.3

A typical wide range out-of-plane scan of XRD for *x* = 0.3 thin films (Fig. 2g) indicates the epitaxial nature of both LSMO and ZrO_2_ on the LAO (001) substrate. The epitaxial relationship of (001)_LSMO_‖(001)_ZrO2_‖(001)_LAO_ are confirmed by the *ф* scan measurements around ZrO_2_ (111) (cubic), ZrO_2_ (202) (cubic), ZrO_2_ (101) (tetragonal) and ZrO_2_ (103) (tetragonal) (Fig. 3). The area ratio fitting was performed according to cubic and tetragonal phase of ZrO_2_ as shown in the inset of Fig. 2g. 62.37% of tetragonal phase and 37.63% of cubic phase for ZrO_2_ are obtained. Combined with the simulated STEM images, this observation of compositional cubic and tetragonal phase displays the interfical reconstruction between ZrO_2_ pillars and LSMO matrix due to cylindrical strain tunning. Hence, the center of pillars is cubic structure while the surface of pillar is tetragonal structure, which is consistent with the out-of-plane compression of ZrO_2_ pillars expected from the mismatch with the LSMO matrix as well. As for strain analysis, with increasing ZrO_2_, the LSMO (002) peak in the (LSMO)_1−*x*_:(ZrO_2_)_*x*_ nanocomposites gradually shifts towards lower angles and becomes sharper compared with the pure LSMO as shown in Fig. 2h, indicating the enhanced strain with increasing *x*. The rocking curves of the (002) peak display a FWHM = 0.26 for *x* = 0.2 and FWHM = 0.22 for *x* = 0.3, illustrating enhanced strain and good sample quality. To understand how this vertical strain is manifested in the (LSMO)_1−*x*_:(ZrO_2_)_*x*_ nanocomposites, the vertical lattice matching (the periodic crystallographic registry) is given (presented in Fig. 2i). The out-of-plane lattice matching is 5 LSMO (001) planes to 4 ZrO_2_ (001) planes. Using our measured lattice parameters (*d*_(001)LSMO_ = 3.93 Å and *d*_(001)ZrO2_ = 5.12 Å for the tetragonal phase), the calculated domain widths for the 5:4 matching in the absence of strain coupling are 19.65 Å and 20.48 Å, respectively, induced by the classic domain matching epitaxy mechanism [38, 39]. This indicates that LSMO is in tension out-of-plane from the vertical ZrO_2_ nanopillars due to smaller *d*_(001)LSMO_ × 5. The vertical strain as calculated form XRD results is 0.77% for *x* = 0.2 and 1.16% for* x* = 0.3, respectively.

**Fig. 4:**
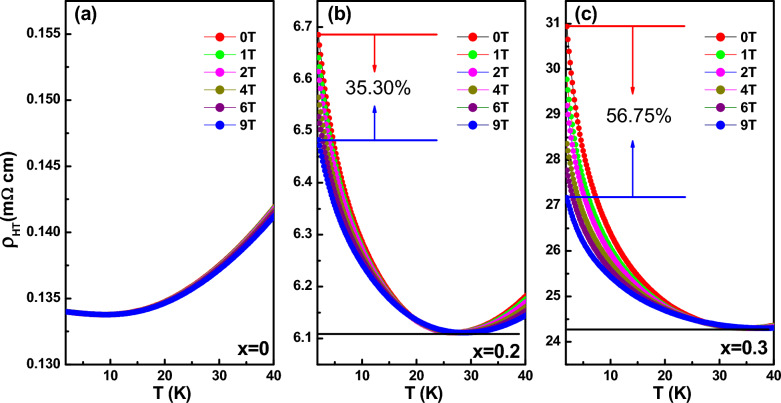
Temperature dependence of resistivity under various magnetic fields applied parallel to the thin film plane for LSMO with *x* = 0 (**a**) 0.2 (**b**) and 0.3 (**c**) respectively. All the data were corrected for the magnetoresistance effect

To elucidate structure–property correlation, the temperature*-*dependent electrical resistivity *ρ*(*T)* under various external *H* for (LSMO)_1−*x*_:(ZrO_2_)_*x*_ is investigated (Fig. 4), where the magnetoresistance (MR) contribution for the resistivity from LSMO here was ruled out by using the way descripted in the Sect. 2. The difference of *ρ*(*T)* between pure LSMO and LSMO with ZrO_2_ pillars are striking. The pure LSMO data displays metallic behavior with negligible field dependence (Fig. 4a). While as temperature decreas, the LSMO with nanopillars display a metal–insulator transition (MIT) at low temperature with a resistivity minimum at the minimum temperature *T*_*min*_ = 27 K for *x* = 0.2 (Fig. 3b) and 36 K for *x* = 0.3 (Fig. 4c), whereas, *T*_*min*_ is slightly dependent of the applied field. The residual resistivity ${\rho }_{0}$ shows two order (6.48 mΩ cm for *x* = 0.2) and three order (27.17 mΩ cm for *x* = 0.3) larger than that of the pure LSMO film (0.13 mΩ cm for *x* = 0). At temperatures below *T*_*min*_, the applied magnetic field suppress the increase of *ρ* indicating a negative effect*,* with increasing field dependence of 35.30% for *x* = 0.2 and 56.75% for *x* = 0.3, respectively. The mechanism of low-*T* MIT here differs from the recently supposed phenomenological model [24] considering the large vertical strain effect as mentioned above from the small ZrO_2_ pillars, which significantly alter the FM behavior of the LSMO matrix [40]. The temperature dependence of *T*_*min*_ and the negative magnetoresistance below *T*_*min*_ display the dominant localization origin. As we evidenced in films containing lower ZrO_2_ previously [32], both weak localization and electron–electron interaction (EEI) contribute the low-*T* MIT. Furthermore, the effect of localization becomes dominant with increasing ZrO_2_ density, this somewhat attenuates the low-*T* EEI contribution sharply as calculated for *x* = 0.03 and 0.06 as comparison [32]. The low-*T* upturn in resistivity from localization is still found even under a 9 T magnetic field at high ZrO_2_ fractions. As discussed by Bergmann [41], the application of a strong magnetic field (≥ 8 T) normaly suppresses WL effect in resistivity, which leaves a temperature dependent resistivity only due to the EEI contribution [41]. In this case, the contributions of WL and EEI can thus be distinguished by using a strong magnetic field to suppress the WL contribution [9], as shown in our previous report [32] for low-density nano-scale ZrO_2_ impurities (where 4 T–6 T is enough to suppress the WL contribution). However, in our present films with high density of second phase of nanopillars (*x* = 0.2 and *x* = 0.3), an even 14 T field is not high enough to suppress the contribution of localization with very high *T*_*min*_. It should be noted that the individual contributions from WL and EEI exclude the possibility that the low-*T* MIT comes from Mott localization. Considering that the localization is tightly correlated with external magnetic field: ^24^, [42] low-dimensional localization is more sensitive to field and has relative larger contribution than EEI at low-*T* region compared with 3D localization. This may be reflected from enhanced field effect with increasing ZrO_2_ content, which gives suppressed resitivity under 9 T field of 35.30% for *x* = 0.2 and 56.75% for *x* = 0.3, respectively as shown in Fig. 4. This enhanced localization we observed in high concentration of the ZrO_2_ nano-pillars.

**Fig. 5:**
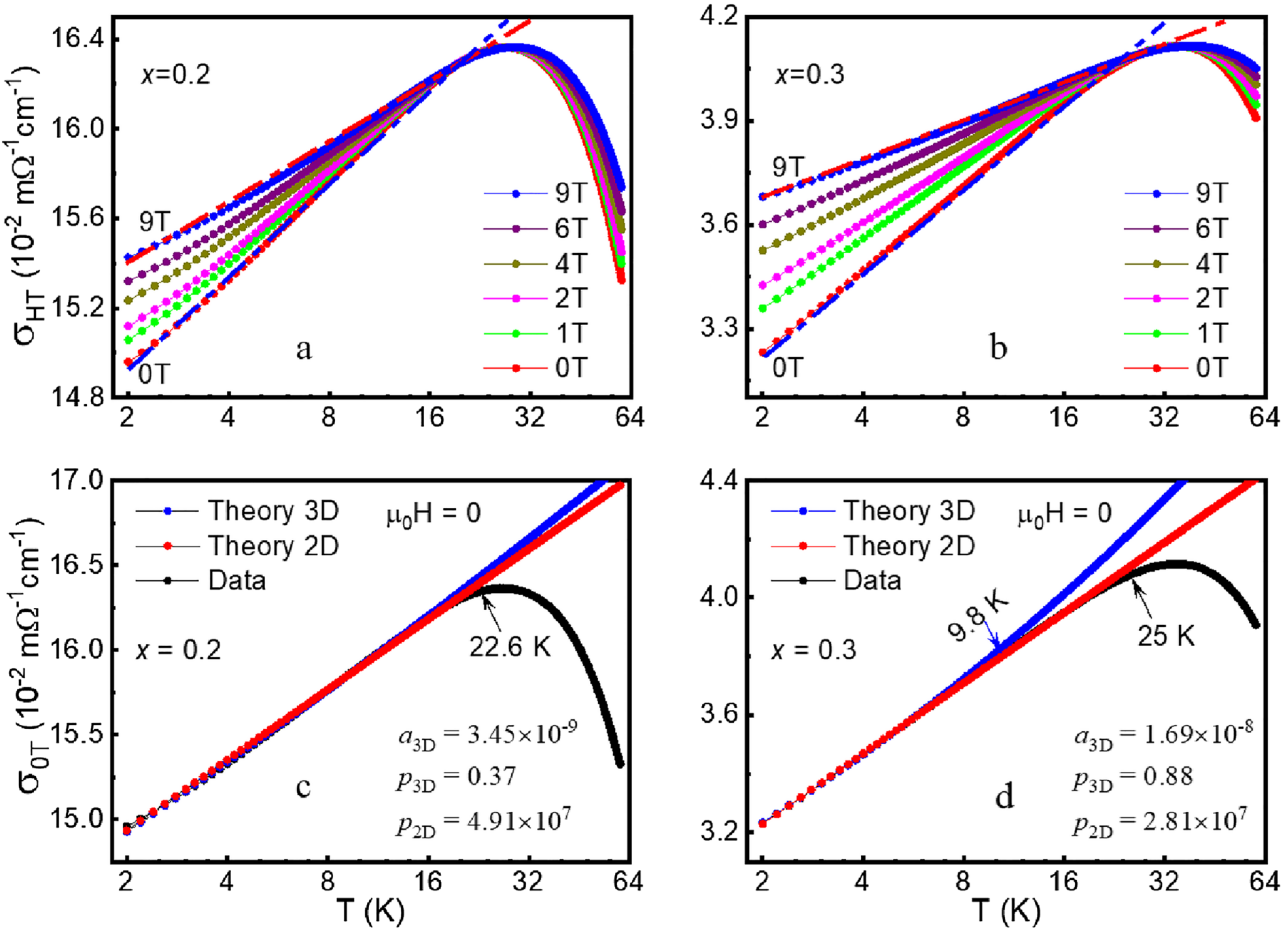
**a,** **b**: Logarithmic temperature dependence of conductivity under various applied magnetic fields parallel to the thin film plane for LSMO with *x* = 0.2 and 0.3, respectively. **c,** **d**: Localization fitting to logarithmic temperature dependence of the $\sigma_{{{\text{0T}}}}$ corresponding to fittings to Eq. (1–3D) and Eq. (1–2D) for *x* = 0.2 and 0.3 thin films, respectively. The fitting parameters are displayed in the inset of the figures

Though 2D localization always exists with any amount of disorder according to scaling theory of localization, [2, 43, 44] 3D localization occurs only above critical level of disorder. To quantify the dimensionality of WL in our present system and reveal the electronic behavior with different strain induced by ZrO_2_ pillars, we analyze our measured electrical conductance according to the scaling theory of localization. In the scalling theory, the behavior of the dimensionless conductance, *g* = *G*/(*e*^2^*/ћ*) is a function of system size *L*. Here *G* is conductance of the films [1, 45]. The scaling function$\beta \left( g \right) = {{d\left( {\ln g} \right)} \mathord{\left/ {\vphantom {{d\left( {\ln g} \right)} {d\left( {\ln L} \right)}}} \right. \kern-0pt} {d\left( {\ln L} \right)}}$ is for various regimes: (1) *g* >> *g*_c_ for large conductance regime (2) *g* << *g*_c_ for small conductance regime, and (3) perturbative regime: *g* ≈ *g*_c_. *g*_c_ is a characteristic dimensionless conductance that turns out to be of order $\pi^{ - 2} \approx 0.1$. We calculated and obtained dimensionless conductance *g* for (LSMO)_1−*x*_:(ZrO_2_)_*x*_: 1707 to 1710 for LSMO, 6.15 to 6.72 for *x* = 0.2, and 1.33 to 1.69 for *x* = 0.3, respectively, from 2 K to *T*_*min*_. Here, *g* is substantially greater than* g*_c_ in the whole system even for the *x* = 0.3 compound, indicating that our samples lie in the large conductance regime. Moreover, *g* rapidly decreases with increasing density of disorder and reaches ~ 1.3 for *x* = 0.3, indicating a tendency to be close to the perturbative limit and near the mobility edge in higher disorder region [43]. Based on one parameter scaling theory [43], *G*_*c*_ ≈ *e*^2^*/ћ* is an unstable fixed point in a 3D system, which signals the mobility edge. To study critical behavior near the mobility edge, one considers predictions of scaling theory for *g*_*0*_ close to *g′*_*c*_ ≈ 1. For *g*_*0*_ > *g′*_*c*_, the distance to the mobility edge is measured by є = *ln*(*g*_*0*_*/g′*_*c*_) ≈ (*g*_*0*_*–g′*_*c*_)/*g′*_*c*_, where *g′*_*c*_ = *G*_*c*_/(*e*^2^*/ћ*), and *g*_*0*_ near *g'*_*c*_. Accoring to our experimental data, an estimation of* g*_*0*_ in the range of 0.28 to 0.52 for *x* = 0.3 was obtained. This broad range of *g*_*0*_ value may reflect the enhanced WL behavior over a broad temperature range in our present system as seen in Figs. 4 and 5.

The logarithmic temperature dependence of electrical conductance *σ*(*T*) is plotted in semi-logarithmic scale for *x* = 0.2 (Fig. 5a) and 0.3 (Fig. 5b). We can see that the logarithmic *T* dependence of *σ* below *T*_*min*_ under various magnetic fields is linear, similar for both compounds. The positive magnetoconductivity shown here illustrates the dominant localization origin. To examine the effective dimensionality of localization, the *σ*(*T*) under *μ*_*0*_*H* = 0 are fitted with the perturbation theory of the singular back scattering [1], which is given by $\sigma_{{{\text{3D}}}} = \sigma_{0} + \frac{{e^{2} }}{{\hbar \pi^{3} }}\frac{1}{a}T^{p/2}$ for 3D localization and $\sigma_{{{\text{2D}}}} = \sigma_{0} + \frac{p}{2}\frac{{e^{2} }}{{\hbar \pi^{2} }}\ln \left( {\frac{T}{{T_{0} }}} \right)$ for 2D localization, respectively [1, 9]. Here, *σ*_*0*_ and *T*_*0*_ are constants and *p* is an index depending on scattering mechanism and dimensionality. The fitting results and fitted parameters (*a* and *p*) according to 3D and 2D scaling theory are shown in Fig. 5c for *x* = 0.2 and Fig. 5d for *x* = 0.3, respectively. It can be seen that the strict one-parameter fitting data both in 2D and 3D cases have a well agreement with the experiment in *x* = 0.2, which suggests that the *x* = 0.2 compound is under the critical condition in dimensionality of WL, i.e., 2D and 3D weak localization are not distinguishable in this system. While for *x* = 0.3, the result shows an excellent agreement with the 2D scenario over a wide *T* range of more than 20K but can only be fitted over a smaller *T* range of around 10K when using the 3D form (Fig. 5d). Thus the electronic behavior in *x* = 0.3 reflects a 2D localization characteristic. To obtain a quantitative understanding to the dimenisional change of electrical behavior in this two compounds, we compare the system size *d* and ${L}_{\varphi }=\sqrt{D{\tau }_{in}}$, [1] Here $L_{\phi }$ represents inelastic scattering length with the diffusion constant $D = \sigma_{0} /[e^{2} N(E_{F} )]$ and the inelastic lifetime *τ*_in_. In the above scale-dependent conductivity $\sigma_{3D} (L_{\phi } )$ and $\sigma_{2D} (L_{\phi } )$ equations, we have taken $\tau_{in} = \frac{{a^{2} }}{D}T^{ - P}$, so $L_{\phi } = aT^{ - p/2}$. Using the well fitted *a* and *p* paramters for both *x* = 0.2 and 0.3 shown in Fig. 5c and d, the characteristic length at *T* = 2 K is *L*_*φ*(*x*=0.2)_ = 3.04 nm and *L*_*φ*(*x*=0.3)_ = 12.46 nm. It is important to mention that the vertical pillars confine the length scale in present system, so *d* is determined by the average shortest distance between pillars, which was obtained as 9.0 ± 0.3 nm for *x* = 0.2 and 6.7 ± 0.3 nm for *x* = 0.3 according to the statistic from the HAADF images (see Fig. 1a). The *L*_*φ*(*x*=0.2)_ = 3.04 nm is comparable to the average pillar distances of* d*_(*x*=0.2)_ = 9.0 ± 0.3 nm, in good agreement with the critical condition of 2D and 3D WL coexistence; While *L*_*φ*(*x*=0.3)_ = 12.46 nm is larger than *d*_(*x*=0.3)_ = 6.7 ± 0.3 nm, reflecting the 2D character and robustness of the WL in *x* = 0.3. These delicate fitting results indicate the competitive 2D and 3D WL in present length scale confinement system, while the 2D WL is more sensitive than 3D WL to the external parameters explaining why low dimensional WL was usually to be experimentally observed as widely studied before.

**Fig. 6:**
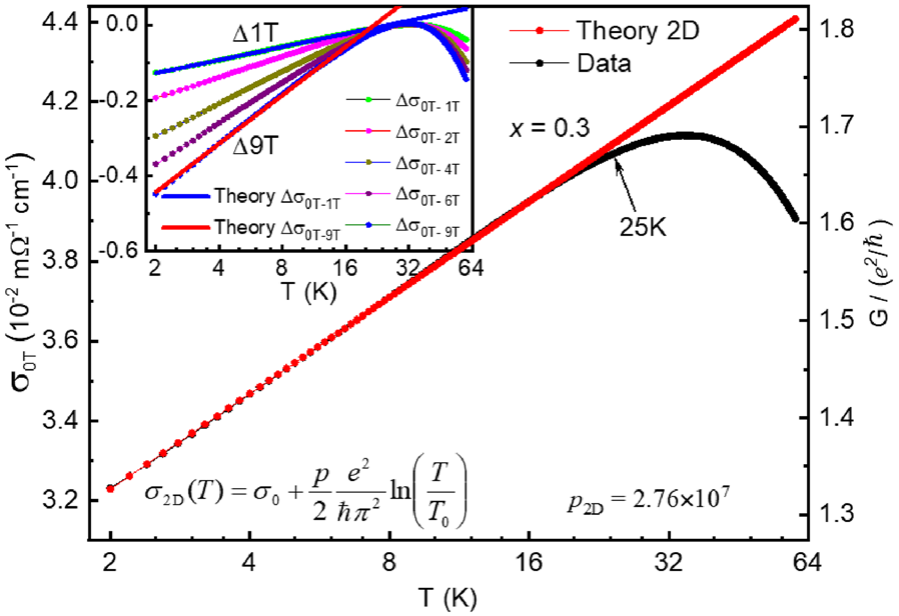
2D Localization theory fitting for the logarithmic temperature dependence of the conductivity ${\sigma }_{0T}$ (left axis) and the dimensionless conductance (right axis) according to Eq. (1–2D) for LSMO samples with *x* = 0.3. Inset: Logarithmic temperature dependence of the contribution of fields on conductivity $\Delta\upsigma ={\sigma }_{0T}-{\sigma }_{HT}$ with applied magnetic fields parallel to the thin film plane of LSMO with *x* = 0.3, along with the localization contribution of fields (2D) fitting to *H* = 1 T and 9 T

Figure 6 emphasizes the good agreement between the conductivity and the strict one-parameter 2D scaling in a wide *T* range of 25 K for *x* = 0.3. Moreover, the field effect on conductivity clearly displays the suppression of WL as indicated by the decreasing *T*_*min*_ with increasing applied field from 1 to 9 T (inset of Fig. 6). In spite of the sensitivity of 2D localization to magnetic field, the WL contribution still dominates under 9 T field applied along in-plane direction, indicating the robust character. Specifically, the pillar-like nanostructures in the films could enhance the multiple scattering quantum interference between waves propagating along the same path but in opposite directions and restrain the amplitude of waves simultaneously, which is the origin of the 2D localization behavior as observed in *x* = 0.3 film. This would suggest strong amplitude fluctuations near the mobility edge corresponding to the lower dimension. The observed enhanced weak localization here, deviating from traditional weak localization, belongs to a new “transition localization” state, which is between weak localization and Anderson localization.

**Fig. 7:**
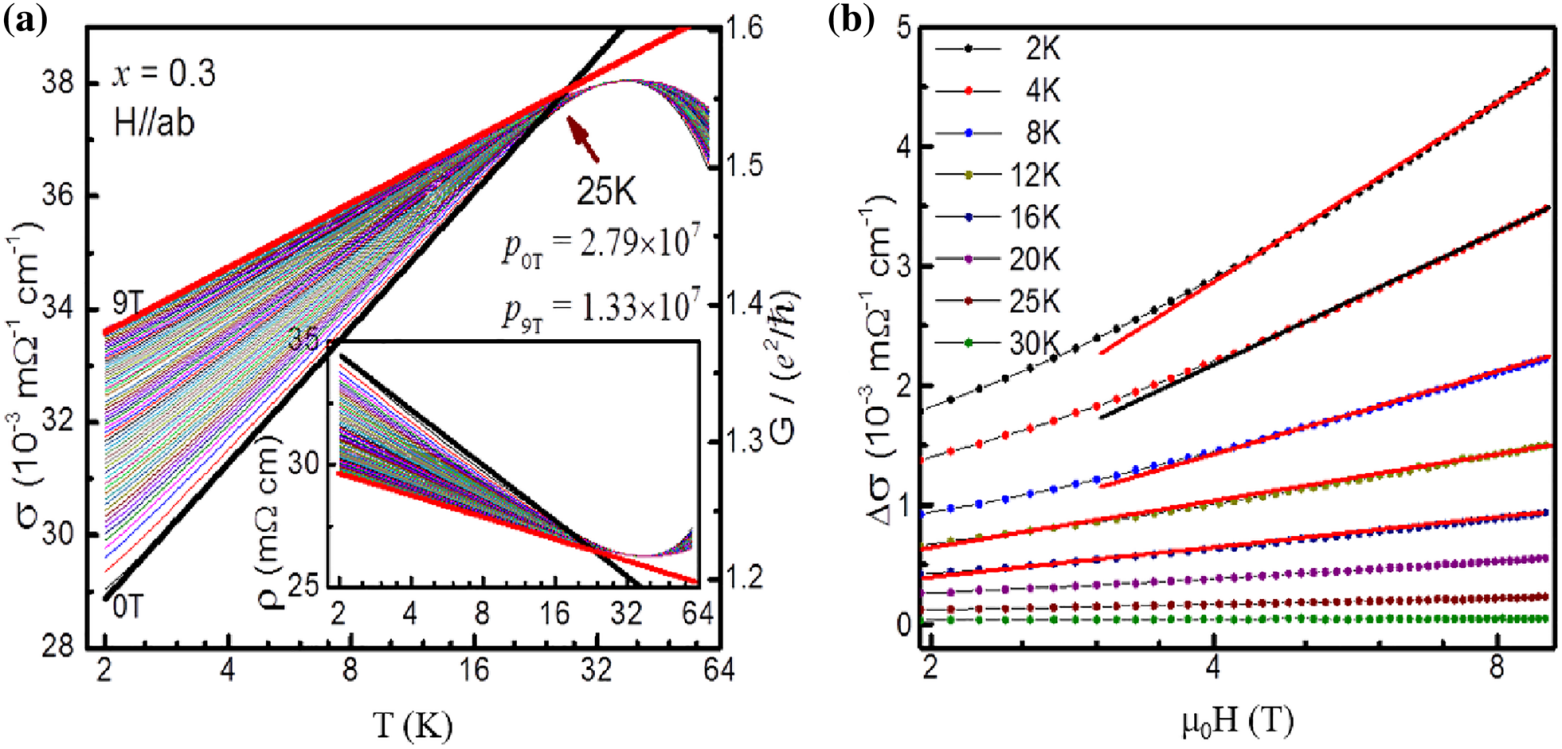
**a** Conductivity σ plotted as a function of *T* between 2 and 55K under various *H*. The Solid line denotes a fit of of the form $\sigma_{{{\text{2D}}}} = \sigma_{0} + \frac{p}{2}\frac{{e^{2} }}{{\hbar \pi^{2} }}\ln \left( {\frac{T}{{T_{0} }}} \right)$ to the data. Inset illustrates the corresponding *ρ* vs *T* under various *H*. **b** $\Delta\upsigma$ as a function of *lnH* at various *T*. The solid line illustrates that $\Delta \sigma$ ∝ *lnH*. The plot is a log-scale plot

**Fig. 8:**
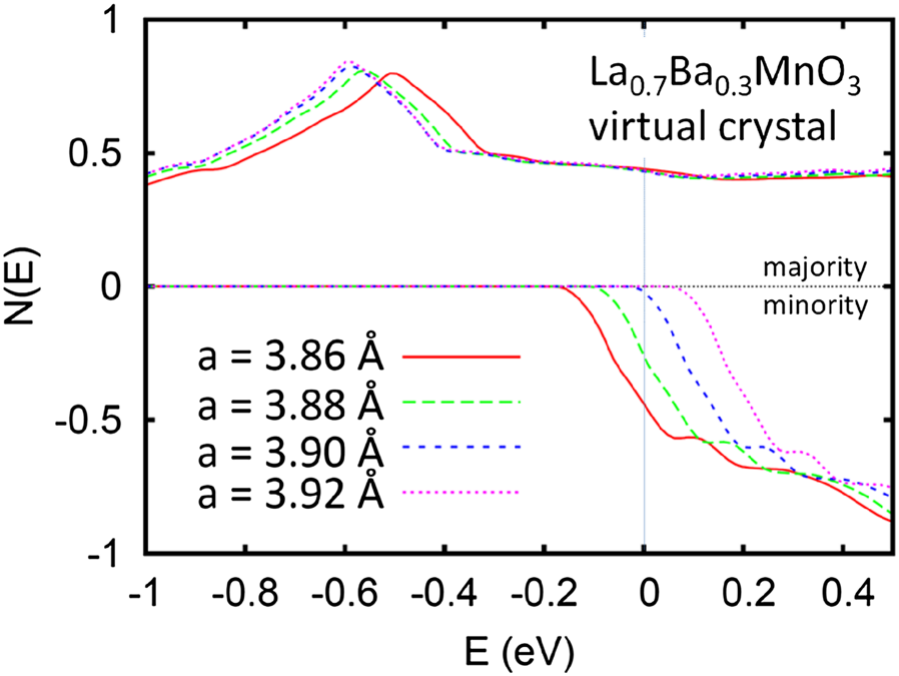
Calculated electronic density of states for cubic perovskite virtual crystal La_0.7_Ba_0.3_MnO_3_ at different lattice parameters as indicated. Note the strong strain dependence of the minority spin band edge with respect to the Fermi level at 0 eV

Further support to the existing 2D localization in *x* = 0.3 is obtained by *T* dependence of *ρ* under various *H* and *H* dependence of *ρ* at various *T* as shown in Fig. 6a, b, respectively. The fitting of *σ*(*T*) to the scaling theory of localization under various magnetic field from 0 to 9 T display good 2D character (Fig. 7a). When apply magnetic field, the cyclotron orbit shrinks until the Landau orbit size *L*_*H*_ << *L*_*φ*_, the predicted 2D formula $\Delta\upsigma =\upsigma \left(H,T\right)-\upsigma \left(0,T\right)=\frac{{e}^{2}}{2{\pi }^{2}\hslash }\left\{\psi \left[\frac{1}{2}+\frac{1}{x}\right]+lnx\right\}$ becomes a *lnH* behavior. Here, *ψ* is the digamma function and $x = L_{\phi }^{2} 4eH/\hbar c$. In this case, the critical minimum field can be determinded. The good agreement of $\Delta \sigma$ versus *H* under different *T* (below *T*_*min*_) with *lnH* behavior as expected illustrates the 2D electrical character in *x* = 0.3 compound (see Fig. 7b). The critical magnetic field *H*_*c*_ varies with *T* as displayed in Fig. 6b. We take 4.4 T (at 2K) as *H*_*c*_, ${L}_{H}={\left(e{H}_{c}/\hslash c\right)}^{-1/2}=12.32 \mathrm{nm}$ can be obtained. This value is comparable to *L*_*φ*(*x*=0.3)_ = 12.46 nm, further evidenced the good 2D electrical character in *x* = 0.3. To trace the effect of tensile strain on LSMO thin film at the interface, we have done virtual crystal calculations with the linearized augmented planewave method using the WIEN2k code and the PBE generalized gradient approximation (Fig. 8). We find that less than 1% tensile strain of La_0.7_Ba_0.3_MnO_3_ converts the transport half metal to a true half metal with no minority carriers, while compressive strain strongly increases the density of localized minority carrier and the effective Fermi level. Thus strains surrounding the ZrO_2_ pillars at the level of 0.77% for *x* = 0.2 and 1.16% for* x* = 0.3 are expected to affect the electronic structure and therefore the electronic scattering of the nano-inclusions, consistent with our experimental results.

## Conclusion

In summary, we have succsusfully synthesized the (LSMO)_1−*x*_:(ZrO_2_)_*x*_ nanocomposite thin films with epitaxial ZrO_2_ pillar structure in the LSMO matrix and investigated the atomic-scale structural, electrical and magnetic properties under the affect of the strained ZrO_2_/LSMO interfaces as spatial disorder. A crosscover from the coexistence of 2D and 3D localization to the typical 2D localization with increasing density and size of ZrO_2_ pillars was experimentally observed, which is interestingly correlated with length scale confinement vertically from the epitaxial pillar-matrix structures. Moreover, the enhanced localization in present systems is identified due to its robustness and enhanced field effect, which is supported by theoretical analysis showing good agreement with one-parameter scaling theory of localization and virtual crystal calculations. Interestingly, based on the excellent agreement of our experimental results with one-parameter scaling theory of localization, the enhanced weak localization exists in metal range close to the fixed point. These films provide a tunable experimental model for studying localization in particular the transition regime by appropriate choice of the second phase. Moreover, our results will facilitate the design of perpendicular magnetic devices.

## Data Availability

Not applicable.

## References

[1] Lee P, Ramakrishnan T (1985). Disordered electronic systems. Rev. Mod. Phys..

[2] Schwartz T, Bartal G, Fishman S, Segev M (2007). Transport and Anderson localization in disordered two-dimensional photonic lattices. Nature.

[3] Hsieh P, Chung C, McMillan J, Tsai M, Lu M, Panoiu N, Wong CW (2015). Photon transport enhanced by transverse Anderson localization in disordered superlattices. Nat. Phys..

[4] Anderson PW (1958). Absence of diffusion in certain random lattices. Phys. Rev..

[5] Datta S (1997). Electronic transport in mesoscopic systems.

[6] Wiersma DS, Bartolini P, Lagendijk A, Righini R (1997). Localization of light in a disordered medium. Nature.

[7] Störzer M, Gross P, Aegerter CM, Maret G (2006). Observation of the critical regime near Anderson localization of light. Phys. Rev. Lett..

[8] Altshuler B, Khmel'Nitzkii D, Larkin A, Lee P (1980). Magnetoresistance and Hall effect in a disordered two-dimensional electron gas. Phys. Rev. B.

[9] Ziese M (2003). Searching for quantum interference effects in La_0.7_Ca_0.3_MnO_3_ films on SrTiO_3_. Phys. Rev. B.

[10] Chang H, Luo J, Wu C, Hsu F, Huang T, Wu P, Wu M, Wang M (2012). Weak localization in FeSe_1−x_Te_x_ superconducting thin films. Supercond. Sci. Technol..

[11] Cao G, Zhang J, Cao S, Jing C, Shen X (2005). Magnetization step, history-dependence, and possible spin quantum transition in Pr 5∕8 Ca 3∕8 MnO3 manganites. Phys. Rev. B.

[12] Pickett WE, Singh DJ (1996). Electronic structure and half-metallic transport in the La_1__−__x_Ca_x_MnO_3_ system. Phys. Rev. B.

[13] Nadgorny B, Mazin I, Osofsky M, Soulen R, Broussard P, Stroud R, Singh D, Harris V, Arsenov A, Mukovskii Y (2001). Origin of high transport spin polarization in La_0.7_Sr_0.3_MnO_3_ direct evidence for minority spin states. Phys. Rev. B.

[14] Pickett W, Singh D (1997). Chemical disorder and charge transport in ferromagnetic manganites. Phys. Rev. B.

[15] Pickett W, Singh D (1997). Transport and fermiology of the ferromagnetic phase of La_2/3_A_1/3_MnO_3_ (A = Ca, Sr, Ba). J. Magn. Magn. Mater..

[16] Llordes A, Palau A, Gázquez J, Coll M, Vlad R, Pomar A, Arbiol J, Guzman R, Ye S, Rouco V (2012). Nanoscale strain-induced pair suppression as a vortex-pinning mechanism in high-temperature superconductors. Nat. Mater..

[17] Gutierrez J, Llordes A, Gazquez J, Gibert M, Roma N, Ricart S, Pomar A, Sandiumenge F, Mestres N, Puig T (2007). Strong isotropic flux pinning in solution-derived YBa_2_Cu_3_O_7−x_ nanocomposite superconductor films. Nat. Mater..

[18] Bruno FY, Garcia-Barriocanal J, Varela M, Nemes N, Thakur P, Cezar J, Brookes N, Rivera-Calzada A, Garcia-Hernandez M, Leon C (2011). Electronic and magnetic reconstructions in La_0.7_Sr_0.3_MnO_3_/SrTiO_3_ heterostructures: a case of enhanced interlayer coupling controlled by the interface. Phys. Rev. Lett.

[19] Yu P, Lee J-S, Okamoto S, Rossell M, Huijben M, Yang C-H, He Q, Zhang J, Yang S, Lee M (2010). Interface ferromagnetism and orbital reconstruction in BiFeO_3_–La_0.7_Sr_0.3_MnO_3_ heterostructures. Phys. Rev. Let..

[20] Yamada H, Ogawa Y, Ishii Y, Sato H, Kawasaki M, Akoh H, Tokura Y (2004). Engineered interface of magnetic oxides. Science.

[21] Garcia V, Bibes M, Barthélémy A, Bowen M, Jacquet E, Contour J-P, Fert A (2004). Temperature dependence of the interfacial spin polarization of La_2/3_Sr_1/3_MnO_3_. Phys. Rev. B.

[22] Hwang H, Iwasa Y, Kawasaki M, Keimer B, Nagaosa N, Tokura Y (2012). Emergent phenomena at oxide interfaces. Nat. Mater..

[23] MacManus-Driscoll JL, Zerrer P, Wang H, Yang H, Yoon J, Fouchet A, Yu R, Blamire MG, Jia Q (2008). Strain control and spontaneous phase ordering in vertical nanocomposite heteroepitaxial thin films. Nat. Mater..

[24] Chen A, Weigand M, Bi Z, Zhang W, Lü X, Dowden P, MacManus-Driscoll JL, Wang H, Jia Q (2014). Evolution of microstructure, strain and physical properties in oxide nanocomposite films. Sci. Rep..

[25] Levin I, Li J, Slutsker J, Roytburd AL (2006). Design of self-assembled multiferroic nanostructures in epitaxial films. Adv. Mater..

[26] Wee SH, Gao Y, Zuev YL, More KL, Meng J, Zhong J, Stocks GM, Goyal A (2013). Self-assembly of nanostructured, complex, multication films via spontaneous phase separation and strain-driven ordering. Adv. Func. Mater..

[27] Zheng H, Wang J, Lofland SE, Ma Z, Mohaddes-Ardabili L, Zhao T, Salamanca-Riba L, Shinde SR, Ogale SB, Bai F, Viehland D, Jia Y, Schlom DG, Wuttig M, Roytburd A, Ramesh R (2004). Multiferroic BaTiO_3_–CoFe_2_O_4_ nanostructures. Science.

[28] Harrington SA, Zhai J, Denev S, Gopalan V, Wang H, Bi Z, Redfern SAT, Baek S-H, Bark CW, Eom C-B, Jia Q, Vickers ME, MacManus-Driscoll JL (2011). Thick lead-free ferroelectric films with high Curie temperatures through nanocomposite-induced strain. Nat. Nanotechnol..

[29] Chen A, Bi Z, Tsai C-F, Lee J, Su Q, Zhang X, Jia Q, MacManus-Driscoll JL, Wang H (2011). Tunable low-field magnetoresistance in (La_07_Sr0_3_MnO_3_)05: (ZnO)05 Self-assembled vertically aligned nanocomposite thin films. Adv. Funct. Mater..

[30] Cao G, Song K, Qiao L, Guo J, Han W, Shen X, Du K, Zhang J, Singh DJ, Gao Y (2021). Vertical strain engineering of epitaxial La_2/3_Sr_1/3_MnO_3_ thin films by spontaneously embedding ZrO_2_ nanopillar arrays. Adv. Mater. Interfaces.

[31] Hoang VV, Cho Y, Yoo JH, Hong S-K, Choi YH, Choi S, Jung W, Jeong CK, Yang J-M (2016). Strain mapping in a nanoscale-triangular SiGe pattern by dark-field electron holography with medium magnification mode. Microscopy.

[32] Gao Y, Cao G, Zhang J, Habermeier H-U (2012). Intrinsic and precipitate-induced quantum corrections to conductivity in La_2/3_Sr_1/3_MnO_3_ thin films. Phys. Rev. B.

[33] Jin San C, Tae Heon K, Chang Won A (2022). 강유전 세라믹의 전기장 인가에 따른 in situ X-선 회절 실험, 전기전자재료학회논문지. J. Korean. Inst. Electr. Electron. Mater. Eng..

[34] Hÿtch M, Snoeck E, Kilaas R (1998). Quantitative measurement of displacement and strain fields from HREM micrographs. Ultramicroscopy.

[35] Hüe F, Hÿtch M, Bender H, Houdellier F, Claverie A (2008). Direct mapping of strain in a strained silicon transistor by high-resolution electron microscopy. Phys. Rev. Lett..

[36] Hirth J, Lothe J (1982). Theory of Dislocations.

[37] Hÿtch MJ, Putaux J-L, Pénisson J-M (2003). Measurement of the displacement field of dislocations to 003 Å by electron microscopy. Nature.

[38] J. Narayan, domain matching epitaxy: a new paradigm for epitaxial growth of oxides, dielectrics for nanosystems: materials science, processing, reliability, and manufacturing: Proceedings of the First International Symposium, The Electrochemical Society, 2004, pp. 103.

[39] Chen C, Lv S, Wang Z, Akagi K, Lichtenberg F, Ikuhara Y, Bednorz JG (2014). Atomic and electronic structure of the SrNbO_3_/SrNbO_3.4_ interface. Appl. Phys. Lett..

[40] Cao G, Song K, Qiao L, Guo J, Han W, Shen X, Du K, Zhang J, Singh DJ, Gao Y (2020). Vertical strain engineering of epitaxial La_2/3_Sr_1/3_MnO_3_ thin films by spontaneously embedding ZrO_2_ nanopillar arrays. Adv. Mater. Interfaces.

[41] Bergmann G (1983). Consistent temperature and field dependence in weak localization. Phys. Rev. B.

[42] Kleinert P, Bryksin V (1995). Anderson localization in anisotropic electronic systems under the influence of weak external electric and magnetic fields. Phys. Rev. B.

[43] Abrahams E, Anderson P, Licciardello D, Ramakrishnan T (1979). Scaling theory of localization: absence of quantum diffusion in two dimensions. Phys. Rev. Lett..

[44] Avishai Y, Luck J (1992). Quantum percolation and ballistic conductance on a lattice of wires. Phys. Rev. B.

[45] Evers F, Mirlin AD (2008). Anderson transitions. Rev. Mod. Phys..

